# Inhibition of sodium‐glucose cotransporter 2 ameliorates renal injury in a novel medaka model of nonalcoholic steatohepatitis‐related kidney disease

**DOI:** 10.1002/2211-5463.12734

**Published:** 2019-11-01

**Authors:** Takuro Nagoya, Kenya Kamimura, Ryo Goto, Yoko Shinagawa‐Kobayashi, Yusuke Niwa, Atsushi Kimura, Norihiro Sakai, Masayoshi Ko, Hiroshi Nishina, Shuji Terai

**Affiliations:** ^1^ Division of Gastroenterology and Hepatology Graduate School of Medical and Dental Sciences Niigata University Japan; ^2^ Department of Developmental and Regenerative Biology Medical Research Institute Tokyo Medical and Dental University Japan

**Keywords:** kidney, medaka disease model, NASH, *Oryzias latipes*, renal disease, SGLT2 inhibitor

## Abstract

The effect of sodium‐glucose cotransporter 2 inhibitor (SGLT2I) on nonalcoholic steatohepatitis (NASH) has been reported, but there are few studies on its effect on NASH‐related renal injury. In this study, we examined the effect of SGLT2I using a novel medaka fish model of NASH‐related kidney disease, which was developed by feeding the d‐rR/Tokyo strain a high‐fat diet. SGLT2I was administered by dissolving it in water of the feeding tank. SGLT2I ameliorates macrophage accumulation and oxidative stress and maintained mitochondrial function in the kidney. The results demonstrate the effect of SGLT2I on NASH‐related renal injury and the usefulness of this novel animal model for research into NASH‐related complications.

## Abstract 2

Here, we demonstrate that the highly specific sodium‐glucose cotransporter 2 inhibitor (SGLT2I) prevented the progression of nonalcoholic steatohepatitis (NASH)‐related renal injury in a medaka fish model of NASH‐related kidney disease. SGLT2I ameliorates oxidative stress and macrophage accumulation and maintained mitochondrial function in renal tubules.

## Graphical abstract

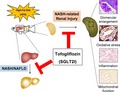

**Abbreviations**
8‐OHdG: 8‐hydroxy‐2’‐deoxyguanosine
GPX1: glutathione peroxidase 1
HFD: high‐fat diet
NAFLD: nonalcoholic fatty liver disease
NASH: nonalcoholic steatohepatitis
OPA1: optic atrophy type 1
SGLT2I: sodium‐glucose cotransporter 2 inhibitor
Tofo: tofogliflozin

Nonalcoholic fatty liver disease (NAFLD) is closely associated with obesity, diabetes, hyperlipidemia, hypertension, and insulin resistance 1, 2, 3 and frequently complicated with various metabolic diseases including chronic kidney disease and cardiovascular disease which can adversely impact prognosis. As insulin resistance is a major risk factor for disease progression, a variety of antidiabetic therapies are expected to provide benefit in NAFLD 4, 5, 6. Among these potential therapeutic approaches, sodium‐glucose cotransporter 2 inhibitors (SGLT2Is), which reduce hyperglycemia by suppressing glucose reabsorption in proximal tubules and improving insulin resistance, glucotoxicity, and lipotoxicity 7, 8, 9, were shown to be effective in ameliorating NAFLD progression in basic 10, 11, 12, 13 and clinical 14, 15, 16, 17, 18, 19, 20, 21, 22, 23, 24 studies. In addition, we recently showed the preventive effect of tofogliflozin (Tofo), a highly specific SGLT2I 13, on fatty infiltration and fibrotic changes in the liver of high‐fat diet (HFD)‐fed medaka fish (*Oryzias latipes*) nonalcoholic steatohepatitis (NASH) model 25. This model provided ease of maintenance of animals under the same condition (the same concentrations of the chemical compound dissolved in water) and high fecundity. Intriguingly, a recent study reported that the HFD‐fed medaka exhibited enlarged glomeruli, dilated glomerular capillaries, and expanded mesangium, changes comparable to changes observed in humans with metabolic syndrome‐related glomerulopathy 26. To date, only a few studies demonstrated the beneficial effect of SGLT2Is on chronic renal injury in NASH animal models and elucidated the underlying mechanisms 27, 28, 29, 30, 31. The mechanisms include the renal inflammation, fibrosis, ER stress, apoptosis and lipid accumulation due to the increased renal expression of reactive oxygen species related to oxidative stress 11, 28 and TGF‐β1, type IV collagen, and fibronectin 11. Therefore, we examined whether NASH‐related renal injury could be ameliorates by the SGLT2I Tofo and assessed renal changes, inflammation, oxidative stress, and renal mitochondrial damage in the medaka model of NASH. We found that Tofo ameliorates the accumulation of macrophages and oxidative stress and maintained mitochondrial function in the renal tubules of medaka fish in the HFD‐induced renal injury model. These results demonstrate the beneficial effect of an SGLT2I in NASH‐related renal injury and further reveal the utility of the medaka model of NASH in examining these effects and evaluation of other potential therapeutic compounds for NASH‐related complications.

## Materials and methods

### Animals and diets

All animal experiments were conducted in full compliance with the regulations of the Institutional Animal Care and Use Committee at Niigata University (Niigata, Japan) that approved the study protocol. All animals received humane care according to the criteria outlined in the ‘Guide for the Care and Use of Laboratory Animals’ by the National Academy of Sciences and published by the National Institute of Health. The d‐rR/Tokyo strain of medaka fish (strain ID: MT837) was supplied by NBRP Medaka (https://shigen.nig.ac.jp/medaka/). All animals were 4 months old and maintained in plastic tanks containing 2 L tap water in plastic tanks under fluorescent light from 8 AM to 8 PM. The water temperature was maintained at 25 ± 1 °C. The medaka NASH model was developed by feeding the fish an HFD described in a previous study 25, 32. Briefly, each tank was supplied with a control diet (Hikari Labo M‐450; Kyorin, Hyogo, Japan) or HFD (HFD32; CLEA Japan, Tokyo, Japan), at a rate of 20 mg·day^−1^ per fish to be consumed within 14 h.

### Tofogliflozin administration

Tofogliflozin (Kowa Co. Ltd., Tokyo, Japan) was prepared at a final concentration of 0.5 mg·L^−1^ in the tank containing the fish to be treated, as described previously 25; this concentration is the *C*_max_ of 500 ng·mL^−1^ in humans treated with a standard Tofo dose of 20 mg. Briefly, Tofo was dissolved in dimethyl sulfoxide (Nacalai Tesque, Kyoto, Japan) to a concentration of 100 mg·mL^−1^ and then added to the water of the plastic tank at a final concentration of 0.5 mg·L^−1^. The same amount of dimethyl sulfoxide was administrated to the tank of the HFD group as vehicle control.

### Histological analyses

Kidney tissue samples were collected at the indicated time points, fixed in 10% formalin, and embedded in paraffin. Sections that were 10 μm in thickness were stained using the standard periodic acid/Schiff method. Mouse anti‐8‐hydroxy‐2′‐deoxyguanosine (8‐OHdG) antibody (ab48508, 1 : 100 dilution; Abcam, Cambridge, UK), rabbit anti‐glutathione peroxidase 1 (GPX1) antibody (ab22604, 1 : 1000 dilution; Abcam), rabbit anti‐F4/80 antibody (ab111101, 1 : 100 dilution; Abcam), rabbit anti‐optic atrophy type 1 (OPA1) antibody (ab157457, 1 : 500 dilution; Abcam), Vectastain Elite ABC rabbit IgG kit (PK‐6101; Vector Laboratories, Burlingame, CA, USA), Vectastain Elite ABC mouse IgG kit (PK‐6102; Vector Laboratories), and DAB chromogen tablets (Muto Pure Chemicals, Tokyo, Japan) were used for immunohistochemical staining. Images were randomly captured from each tissue section, and the measurement of the kidney and glomerular sizes and the quantitative analysis of areas and/or cells stained positively for 8‐OHdG, GPX1, OPA1, and F4/80 were performed using the imagej software (version 1.6.0_20; National Institutes of Health, Bethesda, MD, USA) as reported previously 33. Liver tissue samples were collected at the appropriate time points, fixed in 10% formalin, and embedded in paraffin. Sections (10 μm) were stained with standard hematoxylin and eosin.

### Biochemical analyses

Blood samples were collected from the animals following a 12‐h fasting period for all instances, as previously reported 25. Fish were kept on ice for 1 min; thereafter, they were bled by cutting the ventral portion of the tail fin. Blood was collected in a heparinized microcapillary tube (VC‐H075H; Terumo, Tokyo, Japan) and centrifuged at 1200 ***g*** for 12 min at 22 °C. Blood glucose levels were analyzed using a glucometer (Glucocard G Black, GT‐1830; Arkray, Kyoto, Japan).

### Statistical analyses

Data were analyzed using one‐way or two‐way analysis of variance with repeated measures followed by Bonferroni’s multiple comparison test. A *P* value ≤ 0.05 was considered to indicate statistical significance.

## Results

### Effect of Tofo on blood glucose and kidney size in the medaka model of NASH

**Figure 1:**
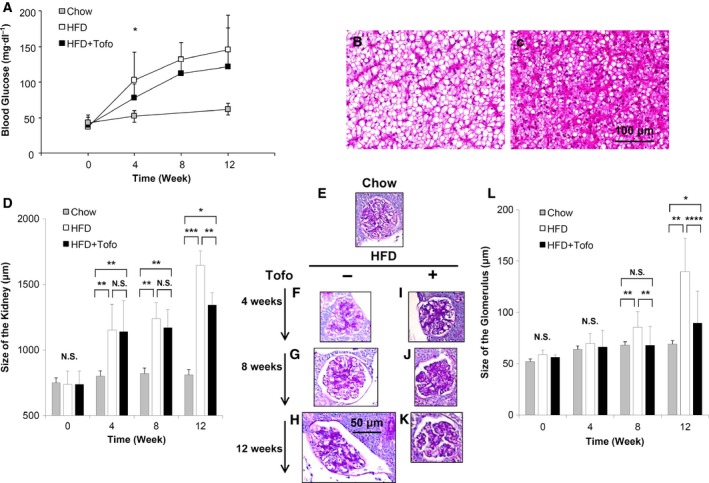
Effects of Tofo on blood glucose, renal size, histological changes, and the glomerular size in the kidney of the medaka model of NASH. (A) The time‐dependent changes in the blood glucose serum biochemical markers. The values represent mean ± SD values (*n* = 15 for each group). Two‐way ANOVA followed by Bonferroni’s multiple comparison test. *P < 0.05. Representative microscopic findings of medaka liver tissues stained with hematoxylin and eosin staining from medaka fed with HFD (B) and HFD + Tofo (C) for 12 weeks. Scale bar represents 100 µm. (D) The time‐dependent changes in the renal size in each group. The values represent mean ± SD values (*n* = 15 for each group). *P < 0.05, **P < 0.01, ***P < 0.001, and NS, no statistical significance. One‐way ANOVA followed by Bonferroni's multiple comparison test. Representative microscopic findings of medaka kidney tissues stained with the periodic acid/Schiff method. (E) Chow‐fed medaka. (F–K) Medaka fed with HFD and HFD + Tofo for 4, 8, and 12 weeks. Scale bar represents 50 µm. (L) Quantitative analysis of glomerular size in the medaka kidneys. The values represent mean ± SD values (*n* = 15 for each group). *P < 0.05, **P < 0.01, ***P < 0.001, ****P < 0.0001, and NS, no statistical significance. One‐way ANOVA followed by Bonferroni’s multiple comparison test.

We first evaluated the effect of Tofo on changes in blood glucose levels over time in chow‐fed (chow), HFD‐fed (HFD), and HFD‐fed and Tofo‐treated (HFD + Tofo) groups (Fig. 1A). Over 12 weeks, whereas blood glucose levels did not show significant changes in the chow group over 12 weeks, the HFD group exhibited a significant increase from the control level of 36.4 ± 4.4 mg·dL^−1^ to 102.8 ± 78.3, 132.2 ± 112.3, and 146.0 ± 121.8 mg·dL^−1^ in 4, 8, and 12 weeks, respectively. Importantly, the blood glucose levels were modestly decreased to 78.3 ± 19.7, 112.3 ± 22.3, and 121.8 ± 54.2 mg·dL^−1^ in 4, 8, and 12 weeks, respectively, in the HFD + Tofo group (*P* < 0.05 in 4 weeks) (Fig. 1A). The liver tissue showed successful development of significant fatty infiltration in the liver (Fig. 1B) and that Tofo ameliorates the changes (Fig. 1C) as previously reported 25. The HFD group exhibited enlargement of the renal size from the control level of 737.7 ± 101.3 µm to 1645.5 ± 109.8 µm following 12 weeks of HFD feeding, whereas the HFD + Tofo group showed milder enlargement to 1343.3 ± 92.5 µm (*P* < 0.01) (Fig. 1D). Mild and statistically nonsignificant changes in renal size were seen over the 12‐week period in the chow group. And the differences between Chow and HFD showed the evidence of successful development of NASH‐related kidney disease. These results confirmed that in the HFD‐induced NASH model in medaka, Tofo was effective in reducing elevated blood glucose level and renal swelling induced by elevated blood glucose.

### Effect of Tofo on glomerular size in the medaka model of NASH

To determine whether glomerular enlargement, a characteristic of HFD‐induced renal injury associated with mesangial expansion and glomerular capillary dilatation, was suppressed by Tofo, we assessed time‐dependent changes in glomerular size in our medaka fish model of NASH. The HFD group exhibited a significant, time‐dependent increase in glomerular size to 140 µm compared with the glomerular size of 60 µm in the chow group over 12 weeks (Fig. 1E–H), and this increase was milder when Tofo was administered (*P* < 0.05) (Fig. 1I–K). No significant changes in the glomerular size were seen in the chow group, and no difference in expansion of the renal matrix was seen in the HFD and HFD + Tofo groups (Fig. 1L). These results suggested that Tofo slowed down HFD‐induced glomerular enlargement in the medaka model of NASH.

### Effect of Tofo on oxidative stress in the kidney of the medaka model of NASH

**Figure 2:**
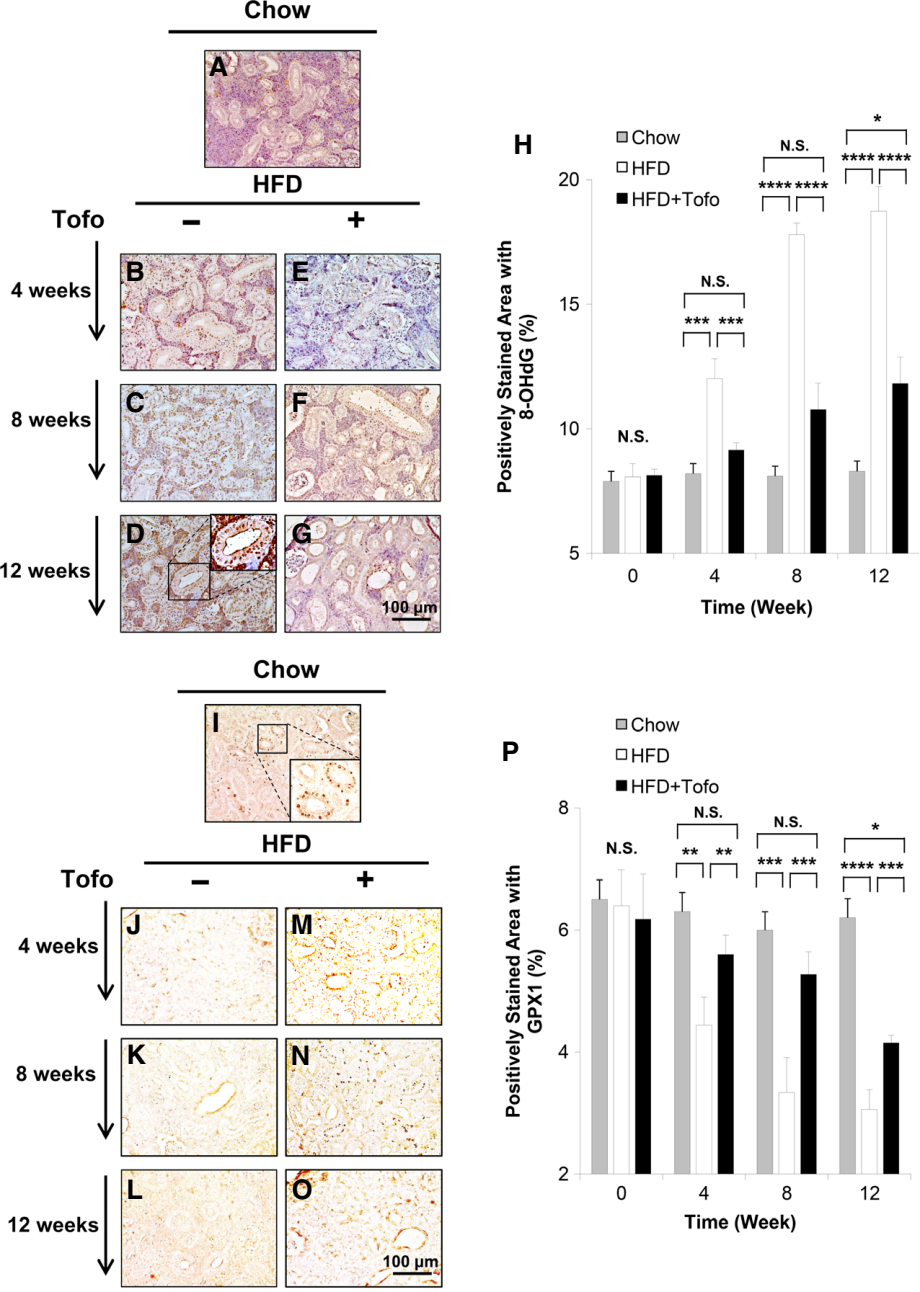
Effects of Tofo on the level of 8‐OHdG and GPX1 in the kidney of the medaka model of NASH. Representative microscopic findings of medaka kidney tissues stained with anti‐8‐OHdG antibody. (A) Chow‐fed medaka. (B–G) Medaka fed with HFD and HFD + Tofo for 4, 8, and 12 week. Scale bar represents 100 µm. (H) Quantitative analysis of positively stained area with anti‐8‐OHdG antibody in the medaka kidneys. The values represent mean ± SD values (*n* = 15 for each group). *P < 0.05, ***P < 0.001, ****P < 0.0001, and NS, no statistical significance. One‐way ANOVA followed by Bonferroni’s multiple comparison test. Representative microscopic findings of medaka kidney tissues stained with anti‐GPX1 antibody. (I) Chow‐fed medaka. (J–O) Medaka fed with HFD and HFD + Tofo for 4, 8, and 12 weeks. Scale bar represents 100 µm. (P) Quantitative analysis of positively stained area with anti‐GPX1 antibody in the medaka kidneys. The values represent mean ± SD values (*n* = 15 for each group). *P < 0.05, **P < 0.01, ***P < 0.001, and NS, no statistical significance. One‐way ANOVA followed by Bonferroni’s multiple comparison test.

To further elucidate the mechanism underlying the effect of Tofo on HFD‐induced renal injury in the medaka model of NASH, we assessed the expression levels of 8‐OHdG and GPX1 in the renal tissues using immunohistochemical analyses (Fig. 2). The HFD group showed increases in the percentage of 8‐OHdG‐positive areas in the renal tissue in a time‐dependent manner, from 8.1 ± 0.5% in the control group (Fig. 2A) to 12.0 ± 0.8%, 17.8 ± 0.5%, and 18.7 ± 1.0% (Fig. 2B–D) at 4, 8, and 12 weeks in the HFD group, respectively. The percentage of 8‐OHdG‐positive areas was significantly suppressed to 9.2 ± 0.3%, 10.8 ± 1.1%, and 11.8 ± 1.1% at 4, 8, and 12 weeks, respectively, in the HFD + Tofo group (Fig. 2E–H). Conversely, the percentage of GPX1‐positive areas was significantly decreased from the control level of 6.4 ± 0.6% (Fig. 2I) to 4.4 ± 0.5%, 3.3 ± 0.6%, and 3.1 ± 0.3% (Fig. 2J–L) at 4, 8, and 12 weeks in the HFD group, respectively, and was significantly suppressed to 5.6 ± 0.3%, 5.3 ± 0.4%, and 4.2 ± 0.1% by Tofo administration (Fig. 2M–P). The differences between Chow and HFD (Fig. 2H,P) suggested that the HFD‐induced renal injury led to oxidative stress represented by the accumulation of 8‐OHdG and the decrease in GPX1‐positive staining, both of which were effectively alleviated by Tofo, although the effect is milder in later stage of 12 weeks.

### Accumulation of F4/80‐positive cells in the kidney of the medaka model of NASH

**Figure 3:**
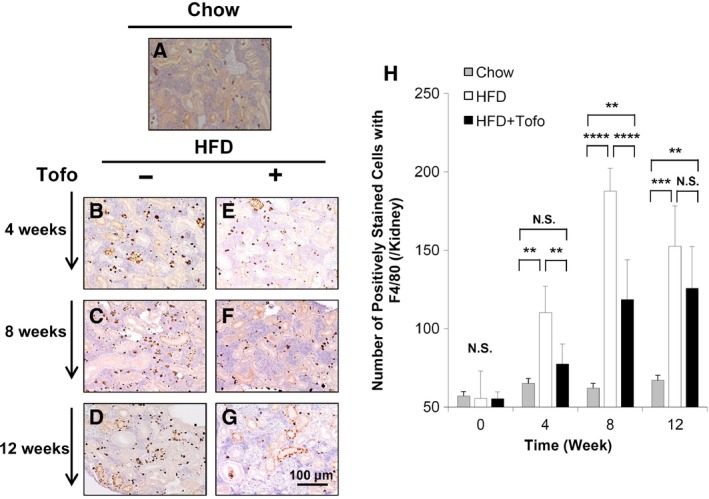
Effects of Tofo on the accumulation of macrophages in the kidney of the medaka model of NASH. Representative microscopic findings of medaka kidney tissues stained with anti‐F4/80 antibody. (A) Chow‐fed medaka. (B–G) Medaka fed with HFD and HFD + Tofo for 4, 8, and 12 weeks. Scale bar represents 100 µm. (H) Quantitative analysis of the number of positively stained cells per high‐power field with anti‐F4/80 antibody in the medaka kidneys. The values represent mean ± SD values (*n* = 15 for each group). **P < 0.01, ***P < 0.001, ****P < 0.0001, and NS, no statistical significance. One‐way ANOVA followed by Bonferroni's multiple comparison test.

To examine the HFD‐induced inflammation in the kidney, we next determined whether there was macrophage accumulation in the kidneys of medaka fish in the HFD‐induced NASH model and whether this could be ameliorates by Tofo. Therefore, we determined the number of cells positively stained with F4/80. While no increase with aging in 12 weeks was observed in the chow group (50–60 cells per high‐power field) (Fig. 3A), the HFD group showed increases in the F4/80‐positive cells over time to 110.1 ± 16.9, 187.6 ± 14.5, and 152.6 ± 25.6 at 4, 8, and 12 weeks, respectively (Fig. 3B–D). This increase was significantly suppressed to 77.5 ± 12.7, 118.5 ± 25.4, and 125.7 ± 26.8 at 4, 8, and 12 weeks, respectively, by Tofo administration (Fig. 3E–H). The differences between Chow and HFD (Fig. 3H) provided evidence that HFD‐induced inflammation and oxidative stress led to an increase in the number of macrophages, which was suppressed by Tofo administration in our animal model although the effect is milder in later stage of 8 and 12 weeks.

### Effect of Tofo on mitochondrial function in renal tubules in the medaka model of NASH

**Figure 4:**
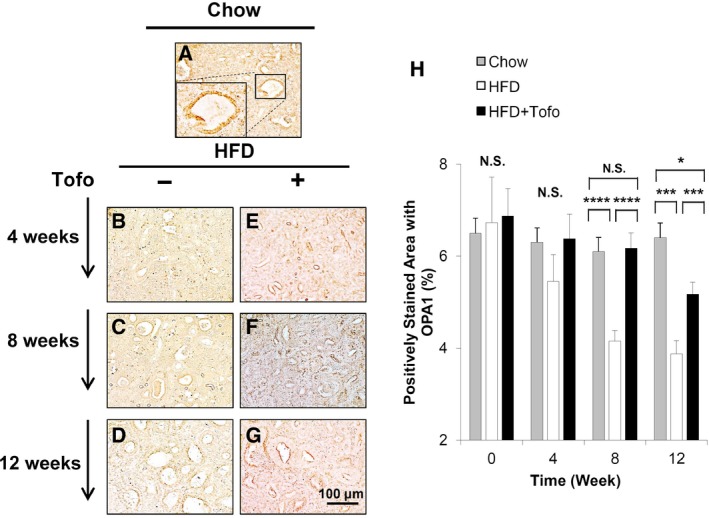
Effects of Tofo on the expression of OPA1 in the renal tubules in the kidney of the medaka model of NASH. Representative microscopic findings of medaka kidney tissues stained with anti‐OPA1 antibody. (A) Chow‐fed medaka. (B–G) Medaka fed with HFD and HFD + Tofo for 4, 8, and 12 weeks. Scale bar represents 100 µm. (H) Quantitative analysis of the number of positively stained cells with anti‐OPA1 antibody in the renal tubules in the medaka kidneys. The values represent mean ± SD values (*n* = 15 for each group). *P < 0.05, ***P < 0.001, ****P < 0.0001, and NS, no statistical significance. One‐way ANOVA followed by Bonferroni's multiple comparison test.

To determine whether HFD led to mitochondrial damage, we assessed the kidney tissue samples in the medaka model of NASH by OPA1 staining. While no change in the percentage of OPA1‐stained areas was observed in the chow group, with 6%–7% OPA1‐positive areas in the control (Fig. 4A), the HFD group exhibited significant decreases in the percentage of OPA1‐positive areas to 5.5 ± 0.6%, 4.2 ± 0.2%, and 3.9 ± 0.3% at 4, 8, and 12 weeks, respectively (Fig. 4B–D). These decreases were reversed with significant increases in the percentage of OPA1‐positive areas to 6.4 ± 0.5%, 6.2 ± 0.3%, and 5.2 ± 0.3% at 4, 8, and 12 weeks, respectively, by Tofo administration (Fig. 4E–H). The differences between Chow and HFD (Fig. 4H) suggested that HFD‐induced renal injury was associated with damaged mitochondrial function in the renal tubules represented by OPA1 expression, a key GTPase necessary for mitochondrial fusion and maintenance of mitochondrial homeostasis, which was suppressed by Tofo administration in our animal model although the effect is milder in later stage of 12 weeks.

## Discussion

With increasing incidence, NAFLD is becoming the most common chronic liver disease. Disease progression is related to obesity, diabetes, hyperlipidemia, hypertension, and insulin resistance 1, 2, 3, and chronic renal injury and cardiovascular disease are common complications that aggravate each other and are associated with bad prognosis in patients with NAFLD/NASH 34. These complications are known to be induced by inflammation and oxidative stress, which aggravate glucotoxicity and lipotoxicity of metabolic syndrome 27, 34. Specifically, NASH‐related renal injury shares several features with diabetic kidney disease and is associated with renal changes including glomerular enlargement, focal segmental glomerulosclerosis, and renal tubular damage 35, 36, 37. The causative factors underlying these features of chronic renal injury include changes in adipokine levels such as a reduction in adiponectin, which mediates fatty acid metabolism by increasing peroxisome proliferative‐activated receptor‐α expression and inducing AMP‐activated protein kinase phosphorylation 38. Basic and clinical studies that provide evidence for these mechanisms used renal histological analyses in HFD‐induced diabetic nephropathy models and reported inflammation, macrophage activation in glomerular as well as interstitial lesions, accumulation of oxidative stress 39, 40, 41, and mitochondrial damage in renal tubules 29, 37. However, few studies have investigated the effect of SGLT2Is on NASH‐related renal injury in an animal model. Therefore, we have examined the renal inflammation, oxidative stress, and mitochondrial function in a medaka model of HFD‐induced renal injury that recapitulates changes in NASH‐associated renal injury 26, because of the ease of maintenance of these animals under the same concentrations of chemical compounds dissolved in water and their high fecundity. In this model, we previously reported that Tofo attenuated the fatty and fibrotic changes in the liver 25. In the current study, we examined the effect of Tofo, a selective SGLT2I, on NASH‐related renal injury by assessing macroscopic and microscopic changes in the kidneys, markers of inflammation and oxidative stress, and mitochondrial function in the renal tubules. Comparison of the Chow and HFD groups with regard to the levels of blood glucose, sizes of the kidneys and glomerulus (Fig. 1), and levels of 8‐OHdG (Fig. 2), GPX1 (Fig. 2), F4/80 (Fig. 3), and OPA1 (Fig. 4) indicated the successful development of NASH‐related kidney diseases. Additionally, after demonstrating the effect of Tofo on blood glucose levels in the medaka model of NASH, we showed that Tofo slowed glomerular enlargement that led to renal swelling induced by HFD. However, our results indicated that Tofo did not affect the mesangial expansion, which is consistent with the findings of a previous report 11, 28, 31. In addition, in our medaka model of NASH, we showed that these changes associated with Tofo administration were related to the amelioration of macrophage accumulation and oxidative stress in the kidney as well as the maintenance of mitochondrial function in the renal tubules in HFD‐induced renal injury in this medaka model of NASH. Our study appeared to have a limitation in the analysis of renal function (including proteinuria and the estimated glomerular filtration rate) because the urinary volume of medaka has been reported to be 1 mL·h^−1^·kg^−1^ and urine is difficult to collect appropriately 42. The comparison of the Chow and HFD + Tofo groups demonstrated that the effect of Tofo became mild with continuous feeding of HFD for 12 weeks, indicating that further analyses regarding the doses and combinations with other antidiabetic drugs, lipid‐lowering drugs, etc., will help improve its effectiveness.

In conclusion, we showed that the highly specific SGLT2I Tofo prevented the progression of NASH‐related renal injury by reducing oxidative stress and macrophage accumulation and maintaining mitochondrial function in the renal tubules. Additionally, the medaka model might be a useful platform for cost‐effective evaluation of the efficacy of therapeutic approaches in NASH‐related complications. Therefore, further analyses involving testing of various medicines in the medaka NASH model will help reveal better combinations of medicines for the treatment and prevention of NASH and its complications.

## Conflict of interest

The authors declare no conflict of interest.

## Author contributions

TS, NH, and KK conceived and supervised the study; KK and NT designed experiments; NT, GR, SKY, NY, KS, SN, KM, and KK performed experiments; and NT, KK, and TS analyzed the data and wrote the manuscript.
